# Multi-omics and their integration in psoriasis research (Review)

**DOI:** 10.3892/mmr.2026.13852

**Published:** 2026-03-23

**Authors:** Hengyan Zhang, Danping Li, Lijuan Zhu, Heguo Yan, Licong Yang, Xuesong Yang, Ye Zhou

**Affiliations:** 1 Department of Dermatology, Zhaotong Hospital of Traditional Chinese Medicine, Zhaotong, Yunnan 657000, P.R. China; 2 Department of Dermatology, Xinping Yi and Dai Autonomous County Traditional Chinese Medicine Hospital, Yuxi, Yunnan 653400, P.R. China; 3 The First School of Clinical Medicine, Yunnan University of Chinese Medicine, Kunming, Yunnan 650500, P.R. China; 4 Department of Dermatology, Yunnan Provincial Hospital of Traditional Chinese Medicine, Kunming, Yunnan 650500, P.R. China

**Keywords:** psoriasis, multi-omics, genome, epigenetics, transcriptome, proteome, metabolome, microbiome

## Abstract

Psoriasis is a chronic, immune-mediated skin disorder characterized by keratinocyte hyperproliferation, inflammatory infiltrates and systemic comorbidities. While genetic predisposition and immune dysregulation are established contributors, recent advancements in high-throughput omics technologies have provided deeper insights into the molecular complexity of psoriasis. The present review synthesized findings from various omics layers, genomics, epigenomics, transcriptomics, proteomics, metabolomics and microbiomics, to elucidate their roles in psoriasis pathogenesis. Large-scale genome-wide association studies have identified both common and region-specific susceptibility loci. Epigenetic factors and transcription factors regulate psoriasis-related genes by modulating chromatin accessibility, DNA methylation, non-coding RNAs and direct gene activation/inactivation, thereby reshaping the transcriptome. Genetic and epigenetic influences also drive significant alterations in the proteome and metabolome, both in the skin and plasma, shedding light on disease mechanisms and offering potential for biomarker discovery. While microbiome research in psoriasis remains in its early stages, shifts in skin and gut microbial communities have been observed, suggesting their involvement in disease pathogenesis. Together, the multi-layered insights underscore the future potential of integrated systems approaches to unravel disease mechanisms and support the discovery of clinically actionable biomarkers and therapeutic targets, paving the way for more precise diagnosis and targeted therapeutic development in psoriasis.

## 1. Introduction

Psoriasis is a chronic, immune-mediated inflammatory disorder primarily affecting the skin, characterized by recurring episodes of erythematous plaques covered with silvery scales, typically on extensor surfaces such as the elbows and knees (1). Clinical variants include palmoplantar, pustular, erythrodermic and guttate types, each presenting with distinct morphological and systemic features (2). Once considered a dermatological condition, psoriasis is increasingly recognized as a systemic disease, with ~75% of patients experiencing at least one comorbidity and a number of affected by multiple concurrent conditions (2).

The pathogenesis of psoriasis is complex and immune-mediated, with high heritability estimated at 60–90% (3). Epidemiological evidence supports this, showing that first- and second-degree relatives of psoriatic patients exhibit markedly higher rates of the disease compared with the general population. Monozygotic twins show even higher concordance rates than dizygotic twins, with ~30% of patients having a first-degree relative also affected (4–6). These findings strongly suggest a heritable basis for psoriasis susceptibility. Extensive research has identified multiple genetic susceptibility loci, further elucidating the genetic architecture of the disease (7).

In addition to genetic predisposition, environmental and epigenetic factors play pivotal roles in disease onset and progression. Modifiable triggers, such as infections, medications, trauma and stress, can influence disease activity via epigenetic modifications, including DNA methylation and histone modifications (8,9). Advancements in high-throughput omics technologies have facilitated integrative analyses of transcriptomic, proteomic and metabolomic data, revealing molecular alterations in psoriatic lesions and highlighting dysregulated pathways as potential therapeutic targets (9–11). Furthermore, emerging evidence uncovers the role of the skin and gut microbiome in modulating immune responses in psoriasis, prompting investigations into host-microbiota interactions as potential contributors to disease pathogenesis and novel therapeutic avenues (12). The present review summarized recent advances in understanding psoriasis pathogenesis, focusing on genetic, epigenetic and systems-level insights that inform improved diagnostic and therapeutic strategies.

## 2. Genetics and epigenetics of psoriasis

### Genetic basis of psoriasis: Recent insights

The substantial heritability of psoriasis highlights a significant genetic component in its pathogenesis (13). Linkage studies, which identify genetic markers co-inherited with the trait within families, have led to the discovery of 15 psoriasis susceptibility loci (PSORS) (14). The primary susceptibility locus, PSORS1 (15), located in the major histocompatibility complex (MHC) on chromosome 6p21.3, accounts for 35–50% of the genetic risk for psoriasis (15,16). The PSORS1 region includes several candidate genes, notably HLA-C (specifically the HLA-C*06:02* allele, also known as HLA-Cw6), CCHCR1 and CDSN (17). HLA-Cw6 is particularly significant as it promotes cytotoxic immune responses in the skin by presenting self-antigens, such as ADAMTS-like protein 5, to CD8^+^ T cells, thereby exacerbating disease onset and severity (18).

The completion of the Human Genome Project and advances in sequencing technologies have greatly expanded genomic datasets, offering valuable resources for investigating genotype-phenotype correlations. Genome-wide association studies (GWAS) using these datasets have markedly advanced our understanding of psoriasis genetics. GWAS have not only confirmed the findings from linkage studies but also facilitated new discoveries. A recent meta-analysis identified 109 distinct PSORS loci, 46 of which had not been previously reported (19). While the number of identified loci continues to increase, it is noteworthy that ethnicity-specific loci have also been identified (20,21). A trans-ethnic GWAS of Caucasian and Chinese populations, for example, revealed 11 population-specific loci (21), prompting recent studies to focus on psoriasis genetics across different ethnic groups and regions (Table I).

### Epigenetics in psoriasis

While genomic factors are central to psoriasis risk, they are not the sole determinants. This is evident from the 64% discordance rate observed among monozygotic twins, who share an identical DNA sequence (22). This suggests that mechanisms beyond genetics contribute to psoriasis pathogenesis. Extensive research has highlighted the critical role of epigenetic factors in psoriasis development (8,23). Epigenetics involves changes in gene expression without alterations to the underlying DNA sequence, typically mediated by DNA methylation, histone modifications and noncoding RNA regulation (24). These epigenetic modifications influence gene expression patterns, further reshaping the transcriptome, proteome and metabolome, all of which contribute to psoriasis. The present review discussed how these epigenetic mechanisms play a role in psoriasis and summarizes recent discoveries in the field.

### DNA methylations

Methylation of cytosines or adenines is a key epigenetic modification that contributes to chromatin repression and gene silencing, playing a significant role in various diseases (25). Typically, DNA methylation in the promoter or enhancer regions of genes is catalyzed by DNA methyltransferases (26). This modification, known as hypermethylation, reduces the binding affinity of transcription factors (TFs) and recruits methyl-CpG binding domain (MBD) proteins such as MeCP2 and MBD2, which help establish a repressive chromatin structure and silence gene expression. Conversely, the removal of methylation marks (hypomethylation), either passively during DNA replication or actively through the action of ten-eleven translocation enzymes, which convert 5-methylcytosine (5-mC) to 5-hydroxymethylcytosine (5-hmC), relieves this repression (26–28). In psoriasis, a self-immune disease, DNA methylation changes have been observed in immune cells. Li *et al* (29) investigated DNA methylation differences in immune cells from the peripheral blood of psoriasis-discordant twins and identified four genes, PTPN6, CCL5, NFATC1 and PRF1, showing differential methylation between psoriasis and unaffected individuals, suggesting their involvement in pathogenesis. In another study focusing on specific cell types, Han *et al* (30) found that naïve CD4^+^ T cells from psoriatic patients exhibited stronger hypomethylation in 26 pericentromeric genomic regions and hypermethylation in the promoter regions of 121 X chromosome genes. While the differential methylation of immune cells has been established, further research is needed to systematically map regulatory targets and gain an improved understanding of their role in psoriasis development (31).

Skin cells, by contrast, exhibit distinct DNA methylation profiles compared with immune cells (32). Specific sites of interest in epidermal cells include hypermethylation of the *p16INK4*a promoter region, observed in ~30% of psoriatic patients. This modification is associated with reduced gene expression and increased keratinocyte proliferation, associated with higher Psoriasis Area and Severity Index scores (33). Hypomethylation, on the other hand, affects loci within the epidermal differentiation complex, such as *S100A7, S100A12* and *OAS2*, leading to enhanced expression of pro-inflammatory genes (34). Additionally, intragenic hypomethylation in the enhancer region of *CYP2S1* has been shown to increase its expression in psoriatic keratinocytes, influencing cell proliferation and immune signaling pathways (35). These methylation alterations disrupt keratinocyte homeostasis and reinforce the chronic inflammatory cycle characteristic of psoriasis (36).

### Histone modifications

Histone proteins undergo various post-translational modifications (PTMs) that influence their interaction with DNA. To date, at least nine distinct types of histone modifications have been identified, with acetylation, methylation, phosphorylation and ubiquitylation being the most extensively studied (37).

In psoriasis, histone methylation and acetylation have been particularly well studied, revealing a distinct histone modification landscape between skin cells and immune cells. In psoriatic keratinocytes, acetylation of Histone H3K27 at the *RPL22* promoter leads to its overexpression, which upregulates Cyclin D1, promotes keratinocyte proliferation, inhibits apoptosis and recruits CD4^+^ T cells (38). Wilms tumor 1 further enhances *IL-1β* expression by increasing histone acetylation in keratinocytes (39). Additionally, Sirtuin 1, a class III histone deacetylase (HDAC) crucial for keratinocyte differentiation, is suppressed by IFN-γ in psoriatic lesions, making the cells more responsive to IL-22 (40). Regarding methylation, reduced levels of H3K9 methylation in keratinocytes are associated with elevated *IL-23* expression, which can trigger IL-17-driven inflammation (41). EZH2, a histone methyltransferase and its product H3K27me3 are both upregulated in the psoriatic epidermis and in keratinocytes stimulated with psoriatic cytokines (42). Inhibition or knockdown of EZH2 markedly reduces keratinocyte proliferation and alleviates psoriasis-like symptoms in mouse models (42). Moreover, CD147 promotes psoriasis onset by modifying H3K9me3 in keratinocytes (43).

Immune cells in psoriasis also exhibit profound changes in histone modifications. In peripheral blood cells, global acetylation of H3 and H4 is reduced in psoriatic patients and inversely correlates with disease severity (44). On the methylation side, increased levels of H3K4me are observed in peripheral blood mononuclear cells from moderate-to-severe plaque psoriatic patients (45). Memory T cells, which are implicated in disease recurrence, display distal H3K27ac at enhancers, supporting persistent transcription of psoriasis-associated genes enriched with GRHL TF-binding motifs (26). Glutaminase 1 enhances H3 acetylation at the *IL-17A* promoter in γδ T17 and Th17 cells, promoting their differentiation and inflammatory activity (46). Furthermore, HDAC activity is essential for the conversion of Tregs into IL-17-producing cells, a process that can be blocked by the HDAC inhibitor trichostatin A (47). While HDAC1 is upregulated in psoriatic skin, SIRT1 is downregulated, reflecting an overall imbalance in histone acetylation in immune cells (48,49). Critically, Jmjd3-mediated H3K27 demethylation regulates Th17 differentiation and its inhibition suppresses IL-17 production by downregulating Th17-related genes (50). Finally, infliximab treatment restores the expression of genes regulated by histone lysine demethylase KDM5B, further supporting a functional link between histone methylation and therapeutic outcomes in psoriasis (51). These cell-type-specific histone modification patterns highlight the divergent roles in psoriasis pathogenesis, with immune cells undergoing changes that affect T cell differentiation, cytokine expression and immune memory, while skin cells primarily exhibit modifications that promote hyperproliferation and cytokine production.

### Non-coding RNAs (ncRNAs)

ncRNAs constitute the majority of the human transcriptome (52). These RNA molecules, which are not translated into proteins, serve diverse regulatory functions by interacting with genes and proteins (53). Well-characterized classes of ncRNAs, including microRNAs (miRNAs), long non-coding RNAs (lncRNAs) and circular RNAs (circRNAs), have been shown to regulate metabolism and homeostasis in both the skin and immune systems. A notable example is the psoriasis susceptibility-related RNA gene induced by stress (PRINS), a lncRNA that is overexpressed in the epidermis of psoriatic patients (54). Further studies indicate that PRINS may play a protective role in cells exposed to psoriatic-related stress. For a comprehensive review of ncRNA studies in psoriasis, readers are encouraged to consult recent publications on miRNAs (55) as well as lncRNAs and circRNAs (56).

## 3. Transcriptional regulation of psoriasis

### Transcription heterogeneity in psoriasis

Transcriptional heterogeneity in psoriasis is evident from the variability in gene expression both between individual cells and across different patients, even within clinically similar lesions (57). This variability contributes to differences in disease severity, lesion characteristics and treatment responses.

Early bulk RNA sequencing (RNA-seq) studies hinted at inter-individual differences in gene expression profiles, suggesting underlying molecular diversity (58,59). More recent advancements in single-cell (sc) RNA-seq have provided detailed insights into transcriptional heterogeneity at a cellular resolution. For example, scRNA-seq analyses of both human and murine psoriatic skin have revealed distinct keratinocyte subtypes, some highly proliferative, others expressing inflammatory genes or undergoing epithelial-to-mesenchymal transition, as well as diverse fibroblast and immune cell populations with unique transcriptional identities (60–62).

This heterogeneity is also apparent within immune cells. scRNA-seq of psoriatic lesions has identified varying activation states of Th17 and tissue-resident memory T cells, with some subsets showing elevated IFN-γ, while others are enriched in chromatin remodeling pathways, highlighting patient-specific immune signatures (60).

Notably, heterogeneity is observed not only between patients or cell types but also within different lesions of the same individual. Non-lesional skin may already exhibit ‘pre-psoriatic’ transcriptional priming and distinct plaques within a single patient can show variable expression of cytokines and inflammatory mediators (60,62–64). This spatial diversity suggests that transcriptional heterogeneity correlates with lesion evolution, location and even therapeutic outcomes.

Another important feature is transcriptional plasticity: Cell states in psoriasis are dynamic, shifting in response to cytokines and targeted therapies. Longitudinal scRNA-seq studies have demonstrated that IL-23 blockade with agents such as risankizumab induces rapid reprogramming of skin cell populations (65,66). Within days, pro-inflammatory fibroblast subsets, specifically WNT5A^+^/IL24^+^ cells, decline sharply, followed by reductions in activated T cells and keratinocytes. By two weeks post-treatment, keratinocyte and myeloid populations exhibit marked downregulation of IL-17/TNF signaling pathways, highlighting the substantial plasticity and reversibility of pathogenic cell states following therapeutic intervention (66).

### TFs in psoriasis

TFs are crucial in regulating gene expression related to immune responses, epidermal differentiation and inflammation, markedly contributing to the transcriptional heterogeneity observed in psoriasis. Transcriptomic analyses of psoriatic skin have revealed differentially expressed genes (DEGs), a number of which encode cytokines and chemokines. Key TFs, such as STAT1, STAT2 and STAT3, have consistently been linked to these DEGs (67–69). For instance, STAT1 is activated in lesional skin of psoriatic patients, where its expression represses IL-22. The imbalance between STAT1 and STAT3 disrupts IL-22 expression, contributing to psoriasis pathogenesis (68,70). STAT2 activates the expression of cytokines CXCL11 and CCL5 in keratinocytes (67). Additionally, lesser-known TFs, such as FOSL1 and FOXC1, have been implicated in regulating psoriasis-associated gene networks (71,72). Notably, FOXC1 negatively correlates with most immune-related DEGs, suggesting its role as a transcriptional repressor in the inflammatory environment of psoriatic lesions (72).

The Th17 immune axis is central to psoriasis pathogenesis, with RORγt acting as the lineage-determining TF for Th17 cells (73,74). RORγt regulates the expression of IL-17A, IL-17F, IL-22 and other pro-inflammatory cytokines that drive epidermal hyperplasia and immune infiltration (75). Inhibition of RORγt by small molecules has emerged as a potential therapeutic approach to simultaneously suppress multiple inflammatory signals (76). The broader balance of T helper cell subsets is also important: psoriatic patients show reduced expression of GATA-3 and IL-4, indicating an impaired Th2 response, while T-bet and IFN-γ expression remain unchanged, reflecting a Th1/Th17-skewed immune profile (77).

Transcriptional regulation in keratinocytes plays a direct role in the pathological features of psoriasis. Dysregulation of the Hedgehog signaling pathway, through increased GLI1 expression, is observed in lesional skin and can be triggered by reduced neurofibromin (NF1) levels. This activation drives keratinocyte proliferation, establishing a mechanistic link between NF1 deficiency and the psoriatic phenotype (78). Other keratinocyte-specific TFs, such as Ovol1 and HES1, influence neutrophil recruitment and the amplification of inflammation, further highlighting the crosstalk between epidermal and immune compartments (79,80).

While NF-κB has been extensively studied in inflammatory pathways, its exact role in psoriasis remains under investigation (69,71). Elevated levels of phosphorylated NF-κB are found in psoriatic skin and therapies such as TNF inhibitors and corticosteroids are thought to partially exert their effects through NF-κB suppression (69). However, due to NF-κB's broad role in immunity, targeting it may require cell-specific strategies to avoid systemic immunosuppression (81). Similarly, metabolic regulators like PPAR-γ are emerging as transcriptional modulators with therapeutic potential. PPAR-γ influences keratinocyte differentiation, skin barrier integrity and inflammation and agonists approved for metabolic diseases may be repurposed to treat psoriatic inflammation and associated comorbidities (82).

Recent advances in single-cell transcriptomics have facilitated the identification of TFs contributing to psoriasis. For example, distinct pathways were activated in different fibroblast cell clusters, governed by specific TFs (83). A recent study using regulon module analysis revealed differential regulon activity between lesional and non-lesional skin in psoriasis, highlighting IRF7 as a key transcriptional regulator. IRF7 was markedly downregulated following guselkumab treatment and its role in modulating the IL-17 pathway highlights its potential as both a disease driver and a therapeutic response marker (84). Collectively, these findings demonstrate that psoriasis is driven by a complex, multi-layered transcriptional hierarchy, where TFs, in conjunction with epigenetic factors, reshape the transcriptomic landscape.

## 4. Protein-level regulation of psoriasis and metabolomics

### Proteins and proteomics in psoriasis

Proteins are central to the pathophysiology of psoriasis, serving not only as structural components of the skin barrier but also as dynamic mediators of inflammation, immune signaling and cellular proliferation. In psoriatic lesions, the dysregulated expression of key proteins, including cytokines such as IL-17A and IL-36γ, chemokines and antimicrobial peptides such as LL37, drives both the initiation and chronic progression of inflammation (85,86). These proteins act as both effectors and biomarkers of immune activation and epidermal dysfunction, making them key targets for mechanistic investigation.

Advances in LC-MS technologies have enabled the development of proteomic studies in psoriasis. Unlike transcriptomics, which captures gene expression potential, proteomics reflects the functional output of cells, including PTMs, protein-protein interactions and secreted mediators (87). Extensive proteomic studies have been conducted on psoriatic patients, primarily using skin and serum samples (88,89).

Human samples and animal models have both been employed in proteomic research, revealing distinct molecular differences between psoriatic and healthy tissues. In murine models, Schonthaler *et al* (90) identified calprotectin as the most upregulated protein in lesional psoriatic skin. Further validation showed that deletion of the S100A9 subunit of calprotectin markedly inhibited psoriasis and inflammation. Human cell lines, such as HaCaT keratinocytes stimulated with TNF-α to model psoriasis, have also been used in proteomics studies. Exosomal proteomes from these models revealed 131 differentially expressed proteins associated with angiogenesis, epigenetic regulation and inflammation (91). Human skin and serum samples further support these findings, with Møller *et al* (92) identifying the inner epidermis as exhibiting the most distinct proteomic alterations, primarily related to innate immunity and cholesterol biosynthesis. Lu *et al* (93) used untargeted proteomics combined with ELISA validation to identify serum pigment epithelium-derived factor as a potential biomarker for diagnosing peripheral psoriatic arthritis (PsA).

Collectively, these proteomic studies provide valuable insights into the pathogenic mechanisms of psoriasis. Interpreting these findings not only facilitates the discovery of novel diagnostic biomarkers but also deepens our understanding of disease-related cellular behaviors at the protein level. Moreover, integrating multi-omics data, such as genomics, transcriptomics and metabolomics, will offer a more comprehensive view of psoriasis' molecular landscape, enabling the identification of key regulatory networks and therapeutic targets (94). For example, by integrating large-scale plasma proteomics with psoriasis GWAS data, Liu *et al* (95) identified AIF1, FCGR3 and HSPA1A as novel, druggable protein targets for psoriasis through proteome-wide Mendelian randomization (MR) and colocalization analysis.

### Metabolomics in psoriasis

The multi-layered genetic regulation of psoriasis leads to a global reshaping of the functional proteome, which alters cellular behavior and, in turn, rewires metabolic processes. This cascade results in a shifted metabolome that reflects the biochemical landscape of the disease. As the most downstream layer of cellular regulation, the metabolome serves as a valuable indicator for understanding the mechanisms of pathogenesis and for identifying diagnostic biomarkers. Beyond their diagnostic value, certain metabolites also play regulatory roles in psoriasis progression. Notably, quinolinic acid, a tryptophan-derived metabolite, has been shown to suppress NOD-like receptor pyrin domain-containing protein 3 inflammasome activation and its supplementation markedly alleviated psoriasiform skin inflammation in murine models (96).

Metabolomic studies in psoriasis have revealed widespread alterations in key biochemical pathways, with consistent findings across various biological samples, including serum, plasma, skin, urine and mononuclear cells. These changes reflect the systemic and localized metabolic disruptions associated with psoriatic disease and offer potential insights for both diagnostic and therapeutic applications.

In serum and plasma, altered levels of amino acids, lipids, carnitines and organic acids have been consistently reported. Amino acids such as arginine, glutamine, cysteine and asparagine show significant variation in psoriatic patients compared with healthy controls (11,97–99). Lipid profiles also exhibit dysregulation, including decreased levels of polyunsaturated fatty acids such as linoleic acid and arachidonic acid, alongside increased lipid peroxidation products (100). Notably, trimethylamine-N-oxide levels correlate with disease severity and comorbidities like cardiovascular conditions (101). Some of these metabolic shifts correlate with treatment response, suggesting a role for metabolomics in monitoring therapeutic outcomes (102).

Skin-targeted metabolomics has provided additional insights into disease-specific metabolic alterations. Lesional skin exhibits altered levels of amino acid derivatives, with concentrations correlating with plaque severity scores (103). A key feature of psoriasis skin is the rewiring of lipid metabolism (104). For example, ceramides, critical metabolites for the skin barrier, are found at altered levels in psoriatic skin compared with healthy tissue. Additionally, elevated concentrations of electrophilic fatty acids and hepoxilins in psoriatic lesions indicate an imbalance in lipid mediator pathways (105,106).

Urinary metabolomics, although less extensively studied, presents an interesting area for research due to the ease of urine collection, making it ideal for diagnostic method development. Researchers have identified potential biomarkers such as reduced citrate levels in PsA patients and elevated tetranor-12(S)-HETE, which reflect neutrophil activity in psoriatic lesions (107,108). Overall, metabolomics studies across different sample types consistently highlight disturbances in amino acid metabolism and lipid pathways in psoriasis. These systemic metabolic shifts may contribute to disease pathogenesis. By integrating proteomic and transcriptomic data, these insights are advancing biomarker discovery for diagnostic purposes (Table II) and will inform the development of therapeutic strategies.

## 5. Microbiome and psoriasis

Microbial organisms at the skin surface interact with the epithelial barrier and immune systems, contributing to the progression of skin barrier diseases (109). In psoriasis, commensal fungi like *Candida albicans* can trigger pathogenic immune responses, particularly Th17 activation and neutrophil recruitment, which directly drive psoriatic skin inflammation (110).

### Skin microbiome

The skin microbiome in psoriasis is marked by reduced microbial diversity and significant compositional shifts compared with healthy skin. Lesional skin exhibits decreased α- and β-diversity, with reductions in key commensals such as *Cutibacterium, Lactobacillus, Burkholderia* and *Corynebacterium*, alongside an increase in potentially pro-inflammatory taxa like *Streptococcus* and *Firmicutes* (111–114). These microbial changes correlate with disease severity, as increased abundance of *Corynebacterium* and *Staphylococcus* has been associated with higher psoriasis severity in some studies (115). However, other research suggests a more varied and less consistent pattern in the psoriatic skin microbiome: While healthy skin is predominantly colonized by *Malassezia* fungi, fungal diversity among psoriatic patients differs and *Malassezia* populations do not appear to differ between healthy and psoriatic skin (116).

### Gut microbiome

Extensive studies have also highlighted the correlation between the gut microbiome and skin barrier diseases, suggesting interactions within the gut-skin axis (117,118). In psoriasis, intestinal barrier biomarkers correlate with disease severity, further supporting the interaction between the skin condition and the gut environment (119,120). Significant alterations in the gut microbiome have been observed in psoriatic patients, though, similar to skin microbiome findings, causality remains unclear. While a number of studies report significant changes in β-diversity (the diversity of microbial communities between samples), most indicate minimal change in α-diversity (the variety of microbes within individual samples) (121). The variability in findings across studies is notable: Some report a decrease in *Bacteroidaceae, Erysipelotrichaceae, Veillonellaceae* and *Bifidobacteriaceae*, while others observe the opposite trend (122–124).

Overall, both the gut and skin microbiomes in psoriasis show significant changes in microbial composition, but current data do not reveal clear patterns. This heterogeneity may stem from variations in study design, such as sampling and sequencing methods, as well as geographic or ethnic differences (125). To address these discrepancies, future research should focus on large, standardized, longitudinal studies with consistent methodology and clear clinical stratification to better define the role of the gut and skin microbiomes in psoriasis.

## 6. Integrating multi-omics for precise diagnosis and targeted therapies for psoriasis

Advances in omics methodologies and the growing body of knowledge in psoriasis have facilitated efforts to bridge discovery and translation in psoriatic diseases by integrating various omics layers to identify biomarkers and therapeutic targets. Methodologies such as MR have enabled the integration of genomic data with other omics datasets, using genetic variants as instrumental variables to infer causal relationships between molecular traits and disease risk. For example, Cai *et al* (94) developed a proteome-wide MR framework by aligning plasma protein quantitative trait loci data with GWAS summary statistics for PsA, identifying seven proteins, NEO1, IL23R, ERAP2, IFNLR1, KIR2DL3, CLSTN3 and POLR2F, that are causally associated with the disease. Significant MR hits were further analyzed through PPI networks to assess connectivity with known drug targets. High-confidence Tier 1 and Tier 2 targets were validated across multiple omics layers, including expression quantitative trait locus (eQTL)-MR for genetic regulation, drug-gene databases for therapeutic tractability, single-cell transcriptomics for tissue specificity and PheWAS for pleiotropic disease relevance, providing insights for potential therapy development (94). Similar MR-based frameworks have been applied in other studies. Notably, Guo *et al* (126) applied MR to integrate DNA methylation, gene expression and protein abundance, identifying a regulatory axis involving lncRNA RP11-977G19.11 and APOF as mediators in psoriasis pathogenesis, thus offering a new potential therapeutic target. They also validated the involvement of TNFAIP3 and MX1, which are critical components of druggable pathways in psoriasis (126).

**Figure 1.**
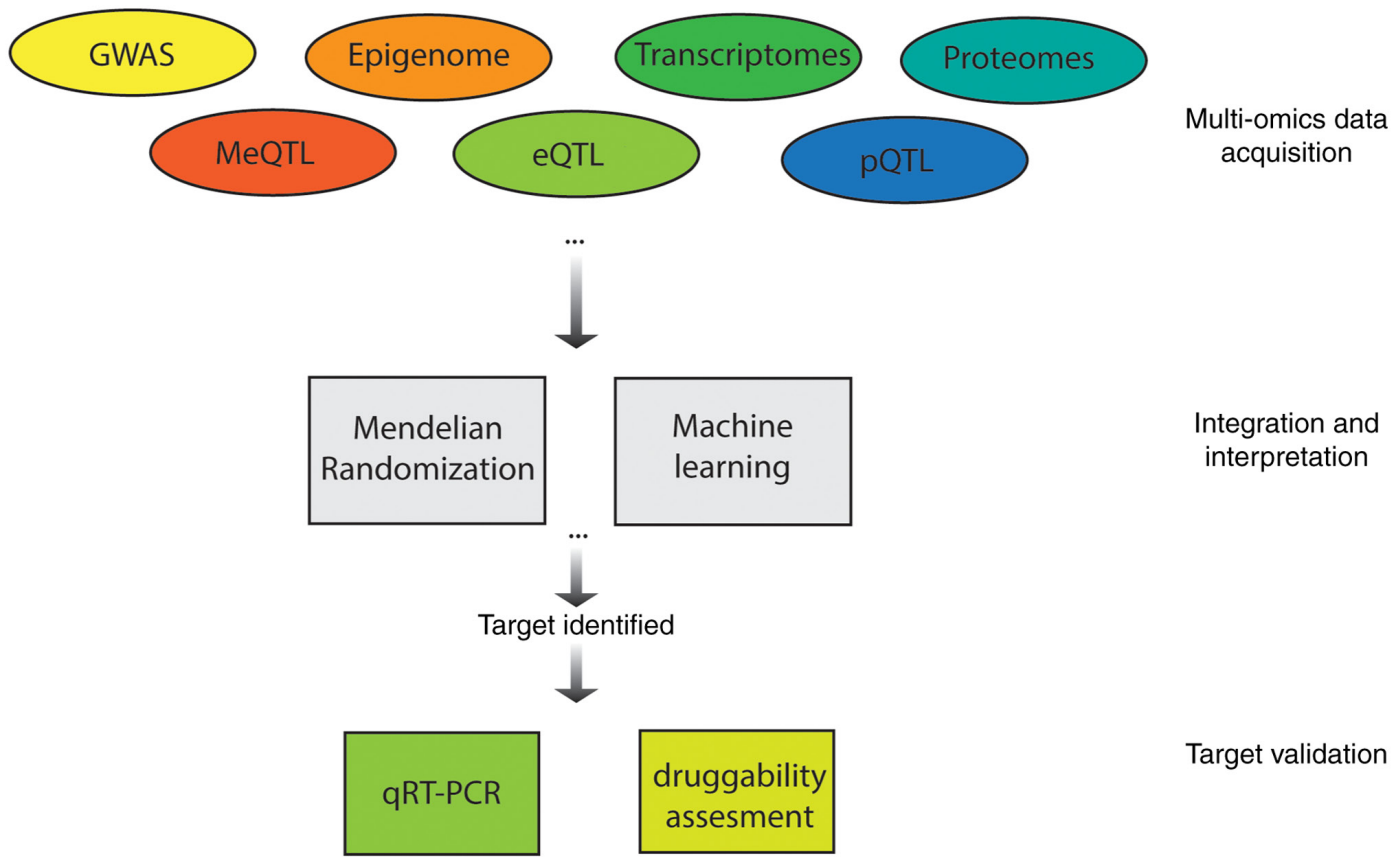
Illustrated workflow of integrating multi-omics data for identifying and validating potential diagnosis and therapeutic targets in psoriasis. Omics datasets, including multi-omics QTL, can be acquired from public databases or experiments and used to capture variations from different layers. These datasets can be integrated using approaches such as Mendelian Randomization and machine learning to identify candidate psoriasis-related targets. Identified targets can be further validated to support their potential use in precision diagnostics and targeted therapy development. MeQTL, methylation QTL; eQTL, expression QTL; pQTL, protein QTL; QTL, quantitative trait locus.

Machine learning has also emerged as a powerful tool in multi-omics-based psoriasis research. For instance, Xing *et al* (127) combined transcriptomic and DNA methylation datasets from psoriasis lesions and controls to identify differentially expressed methylated genes. Machine learning algorithms were then used to pinpoint GJB2 as a hub gene with strong diagnostic value, which was validated through reverse transcription-quantitative PCR and immunohistochemistry. In parallel, Deng *et al* (128) applied a multi-omics approach integrating serum proteomics and skin transcriptomics, alongside machine learning classification, to identify PI3 as a psoriasis biomarker linked to disease severity and keratinocyte hyperproliferation (128). Integrating data across multiple omics layers offers a more comprehensive understanding of disease mechanisms and facilitates the discovery of robust biomarkers and therapeutic targets (Fig. 1). Continued advances in data integration methods will be critical for translating multi-omics insights into biomarkers for precision diagnostics. Moreover, robust models for target-guided drug screening will help validate multi-omics findings and support the development of effective psoriasis treatments (129).

## 7. Conclusion and future perspectives

**Figure 2.**
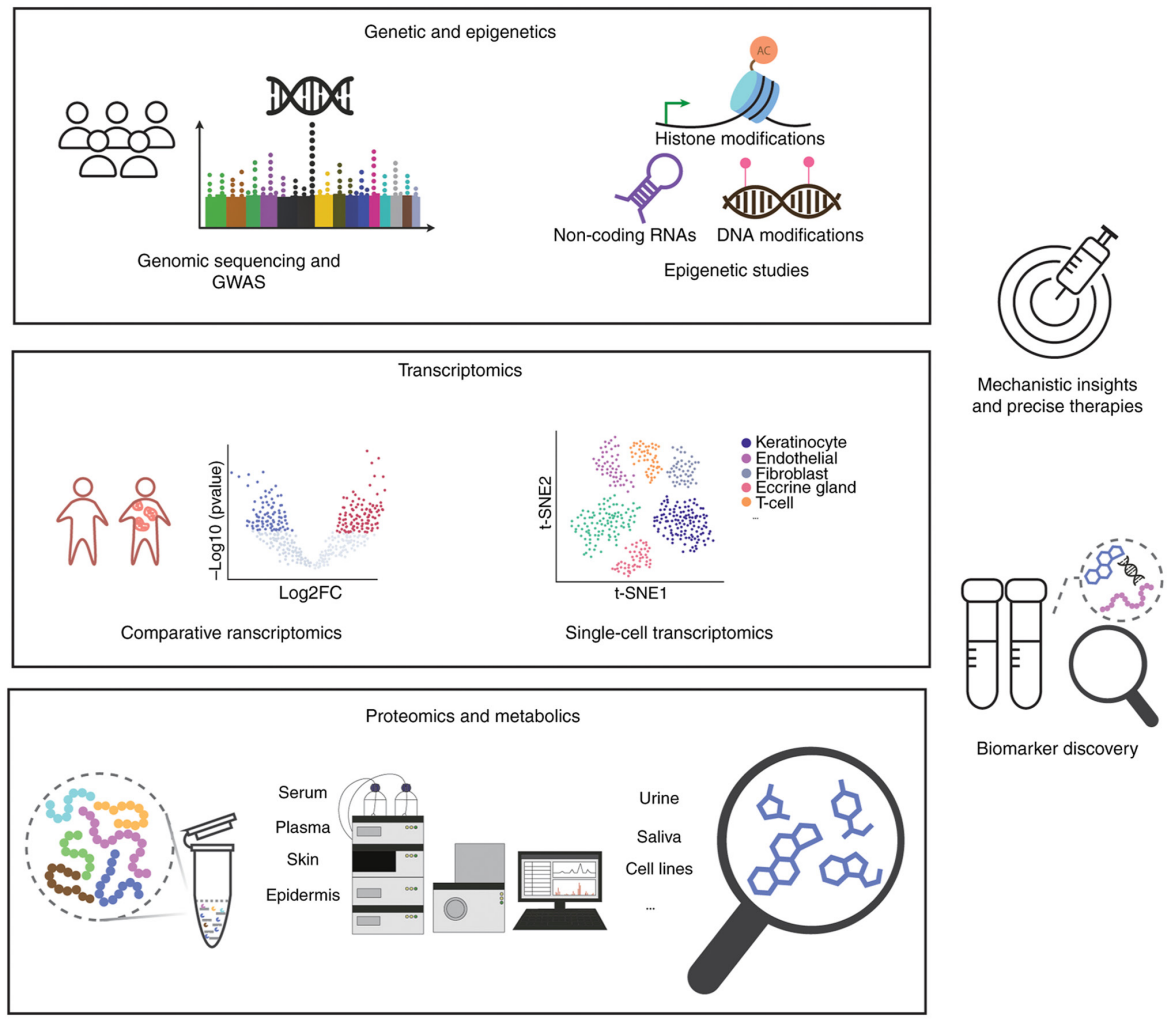
The multi-omics landscape of psoriasis research. The development of technologies in genetics and epigenetics, transcriptomics, proteomics and metabolomics generates key data that can be integrated to study psoriasis mechanisms for precise therapy, as well as identify biomarkers for diagnosis. GWAS, genome-wide association studies.

Traditionally regarded as an immune-mediated disease, psoriasis pathogenesis has been broadened by multi-omics investigations, extending beyond immune cell activation and keratinocyte proliferation, emphasizing the roles of complex immune signaling, transcriptional heterogeneity, dynamic proteomic changes, metabolic rewiring and host-microbiome interactions. These multi-layered insights not only enhance our understanding of disease mechanisms but also identify promising biomarkers and therapeutic targets (Fig. 2). In the future, integrating omics data across spatial, temporal and cellular dimensions will be crucial for advancing precision diagnostics and targeted therapies.

Despite these advances, several challenges remain in translating multi-omics findings into clinical applications. First, at the single-omic level, inconsistencies in findings across studies are prevalent, especially in microbiome and metabolome research, where results often vary due to technical differences, limited sample sizes, lack of longitudinal data and heterogeneity of patient cohorts. These inconsistencies hinder the establishment of robust biomarkers and call for standardized experimental protocols and reference datasets.

Second, integrating multiple omics layers introduces its own limitations. Although computational integration methods, such as MR and machine learning, have enhanced target identification, inconsistent data quality and the lack of open-access, harmonized datasets may limit reproducibility and cross-study comparability. Moreover, a number of current studies suffer from limited sample sizes, particularly when combining multi-omics data from the same individuals, which reduces statistical power and increases the risk of overfitting in computational models. Small and imbalanced datasets can also obscure subtle biological signals, limiting the generalizability of findings across diverse patient populations. Future endeavors should prioritize the generation of large, well-annotated, multi-omics cohorts with matched clinical metadata, as well as the development of standardized pipelines and open-access platforms to facilitate reproducibility, benchmarking and collaborative discovery in psoriasis research.

Meanwhile, it is noteworthy that a number of computationally identified biomarkers and therapeutic targets remain unvalidated. A number of studies stop at correlation-based insights without performing *in vitro* or *in vivo* functional assays to establish causality or druggability, creating a translational bottleneck. Bridging this gap will require robust experimental follow-up and the integration of clinical metadata to contextualize findings.

In conclusion, while multi-omics approaches have shown great promise in unraveling the molecular complexity of psoriasis, future efforts should prioritize methodological standardization, cross-layer validation and translational research to fully realize the potential of these insights in precision diagnostics and targeted therapies.

## Figures and Tables

**Table I.** Selected summarization of recent studies on GWAS of psoriasis in different ethnic groups/regions.

First author/s, year	Ethnic group/Region	Sample size (patient/control)	Result	(Refs.)
Zhang *et al*, 2009	Chinese Han and Uyghur ancestry	1139/1132 and 539/824	Identified a new susceptibility locus within the *LCE* gene cluster on 1q21	(20)
Tamari *et al*, 2014	Japan	999/938	Highlighted the importance of TNIP, *IL12B, TRAF3IP2* loci and the MHC class I region in the Japanese population	(130)
Nasser *et al*, 2025	Egypt	600/600	Identified novel genome-wide significant associations in the HLA region and near the IL23R gene	(131)
Yang *et al*, 2024	Taiwan	2248/67440	HLA-A*02:07 and HLA-C*06:02 markedly contribute to psoriasis	(132)
Ellinghaus *et al*, 2010	Germany	472/1146	Identified an association at *TRAF3IP2* on 6q21	(133)

GWAS, genome-wide association studies.

**Table II.** Recently reported biomarkers for psoriasis.

A, Genes				

First author/s, year	Biomarker(s)	Sample type	Method for biomarker discovery	(Refs.)
Xing *et al*, 2022	*GJB2*		DEGs and DMR-genes between psoriasis and control samples were combined to obtain differentially expressed methylated genes	(127)
Deng *et al*., 2023	*PI3*	Serum	Proteome profiling, transcriptome sequencing and single-cell transcriptome were performed	(128)
Zhou *et al*, 2024	*CCNE1*	Lesional skin	Machine learning algorithms were applied to screen diagnosis markers	(134)
Yang *et al*, 2022	*CXCL8*	Lesional skin	Protein-protein interaction network analysis showed that CXCL8 was the novel hub gene of psoriasis and associated with 22 types of infiltrating immune cells	(135)

**B, Proteins**				

**First author/s, year**	**Biomarker(s)**	**Sample type**	**Method for biomarker discovery**	**(Refs.)**

Sikora *et al*, 2019	Intestinal fatty acid binding protein	Serum	Serum concentration of I-FABP was measured using a commercially available ELISA kit	(119)
Mao *et al*, 2024	MMP12, PCSK9, PRSS8 and SCLY	Blood	Multivariate Cox regression models were used to investigate serum proteins and their relations to psoriasis risks. This was followed by two-sample Mendelian randomization	(136)
Zhou *et al*, 2020	OAS2	Epidermis and serum	Quantitative proteomics analysis by iTRAQ labeled LC-MS/MS	(137)

**C, Metabolites**				

**First author/s, year**	**Biomarker(s)**	**Sample type**	**Method for biomarker discovery**	**(Refs.)**

Kang *et al*, 2017	20 metabolites related to glycolysis pathway and amino acid metabolism	Serum	Multivariate statistical analysis of metabolomics data	(97)
Alonso *et al*, 2016	Citrate	Urine	Untargeted metabolomics and multipletest correction	(108)
Song *et al*, 2023	The combination of 10 differential metabolites	Plasma	All differential metabolites from untargeted metabolomics were used for univariate ROC analysis and metabolites with an AUC ≥0.8 were selected as potential biomarker candidates	(138)

CXCL8, C-X-C motif chemokine ligand 8; DEGs, differentially expressed genes; DMR, differentially methylated region.

## Data Availability

Not applicable.
